# Pkh1p-Ypk1p and Pkh1p-Sch9p Pathways Are Activated by Acetic Acid to Induce a Mitochondrial-Dependent Regulated Cell Death

**DOI:** 10.1155/2020/7095078

**Published:** 2020-04-02

**Authors:** António Rego, Filipa Mendes, Vítor Costa, Susana Rodrigues Chaves, Manuela Côrte-Real

**Affiliations:** ^1^Departamento de Biologia, Centro de Biologia Molecular e Ambiental, Universidade do Minho, Braga, Portugal; ^2^Instituto de Investigação e Inovação em Saúde, Universidade do Porto, Porto, Portugal; ^3^Instituto de Biologia Molecular e Celular, Universidade do Porto, Porto, Portugal; ^4^Departamento de Biologia Molecular, Instituto de Ciências Biomédicas Abel Salazar, Universidade do Porto, Porto, Portugal

## Abstract

The yeast *Saccharomyces cerevisiae* undergoes a mitochondrial-dependent regulated cell death (RCD) exhibiting typical markers of mammalian apoptosis. We have previously shown that ceramide production contributes to RCD induced by acetic acid and is involved in mitochondrial outer membrane permeabilization and cytochrome *c* release, especially through hydrolysis of complex sphingolipids catalyzed by Isc1p. Recently, we also showed that Sch9p regulates the translocation of Isc1p from the endoplasmic reticulum into mitochondria, perturbing sphingolipid balance and determining cell fate. In this study, we addressed the role of other signaling proteins in acetic acid-induced RCD. We found that single deletion of *PKH1* or *YPK1*, as shown for *SCH9* and *ISC1*, leads to an increase in cell survival in response to acetic acid and that Pkh1/2p-dependent phosphorylation of Ypk1p and Sch9p increases under these conditions. These results indicate that Pkh1p regulates acetic acid-induced RCD through Ypk1p and Sch9p. In addition, our results suggest that Pkh1p-Ypk1p is necessary for *isc1Δ* resistance to acetic acid-induced RCD. Moreover, double deletion of *ISC1* and *PKH1* has a drastic effect on cell survival associated with increased ROS accumulation and release of cytochrome *c*, which is counteracted by overexpression of the PKA pathway negative regulator *PDE2*. Overall, our results suggest that Pkh1p-Ypk1p and Pkh1p-Sch9p pathways contribute to RCD induced by acetic acid.

## 1. Introduction

Sphingolipids are essential components of all eukaryotic membranes. In addition to their structural function, sphingolipids play important roles in cellular responses to many different stimuli, acting as messengers in a variety of signaling pathways. From yeast to higher eukaryotes, sphingolipids are involved in the regulation of cell growth, apoptosis, cell cycle arrest, cell wall integrity, nutrient uptake, and longevity [1, 2]. Moreover, sphingolipids have been associated with several human pathologies linked to apoptosis dysfunction, such as cancer and neurodegenerative diseases [3]. Consequently, understanding how signaling pathways regulate sphingolipid metabolism and cell death will provide new hints to the elucidation of molecular mechanisms underlying these diseases.

Sphingolipid metabolism is highly complex and interconnected and its regulation is of fundamental importance for cells due to their relevant role in cellular homeostasis [4]. Therefore, cells possess several mechanisms that contribute to the control of enzymes at different steps of sphingolipid synthesis and breakdown to adjust the levels of individual sphingolipids. However, in response to stress conditions, many biosynthetic intermediates can unduly accumulate and severely influence cell functions and fate. Based on several observations, it is now established that sphingolipids play a role in apoptosis [5]. Exposure of yeast cells to acetic acid has been shown to trigger a regulated cell death (RCD) process with features similar to mammalian apoptosis, such as exposure of phosphatidylserine to the outer leaflet of the plasma membrane, alterations in mitochondrial structure, chromatin condensation, nuclear DNA fragmentation, caspase-like activation, reactive oxygen species (ROS) accumulation, cytochrome *c* release, and mitochondrial dysfunction [6–10]. An increase in ceramide levels during apoptosis has been also reported in response to a variety of stimuli and in different cell types. In particular, we have reported the involvement of ceramide metabolism in cell death induced by acetic acid. We showed that *isc1Δ* and *lag1Δ* mutants, lacking inositol phosphosphingolipid phospholipase C and ceramide synthase, respectively, exhibited a higher resistance to acetic acid that was associated with a decrease in some dihydroceramide and phytoceramide species. Accordingly, these mutants also displayed lower levels of ROS accumulation and reduced mitochondrial alterations, which led us to conclude that acetic acid increases ceramide levels through hydrolysis of complex lipids and *de novo* synthesis catalyzed by Isc1p and Lag1p, respectively, leading to a mitochondria-mediated RCD [11]. However, several important questions related to this model system remained to be elucidated, including which signaling pathways are involved in the activation of these enzymes in the course of acetic acid-induced cell death.

**Figure 1:**
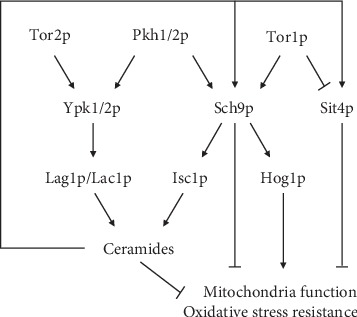
Schematic overview of the signaling pathways involved in sphingolipid metabolism, mitochondrial function, and oxidative stress resistance. Ypk1p is the sole paralog responsible for the phosphorylation of ceramide synthases Lac1p and Lag1p, establishing the link between this kinase and sphingolipids [26]. Sch9p functions as the major gatekeeper of sphingolipid homeostasis, as a feedback system complementary to ceramide production in response to both internal sphingolipid signals and nutrient availability and for the proper translocation of Isc1p from the ER to mitochondria during the diauxic shift [27]. Recently, we showed that Sch9p modulates acetic acid-induced mitochondrial cell death through the regulation of Isc1p cellular distribution, thus affecting the sphingolipid balance and ultimately regulating cell fate [28].

An important regulator of the yeast sphingolipid biosynthetic pathway, which controls the sphingolipid balance, is the serine/threonine kinase Pkh1p (Figure 1). Pkh1p, and its paralog Pkh2p, is an orthologue of the mammalian 3-phosphoinositide-dependent kinase PDK1 [12]. Four protein kinase substrates of Pkh1p and/or Pkh2p have already been identified as Pkc1p [13, 14], Ypk1p, Ypk2p, and Sch9p [12, 15]. In particular, the serine/threonine protein kinases Ypk1/2p, orthologues of the mammalian protein kinase serum- and glucocorticoid-inducible protein kinase (SGK), are phosphorylated and activated by Pkh1/2p; Pkh1p preferentially activates Ypk1p and Pkh2p preferentially activates Ypk2p [16]. Ypk1p activation also requires its phosphorylation by the Target of Rapamycin (TOR) complex 2 (TORC2) [17]. Sch9p can be phosphorylated by Pkh1/2p at the PDK1 site in its activation loop [18, 19] or by TORC1 (TOR complex 1) at multiple C-terminal sites [20]. A recent work showed that Sch9p kinase is also necessary for yeast to properly adapt to hyperosmotic stress, at least in part, through the Hog1p MAPK complex [21]. In yeast, Sit4p is the catalytic subunit of the ceramide-activated protein phosphatase (CAPP) [22]. This phosphatase plays a key regulator role in sphingolipid metabolism by acting downstream of TORC1 signaling. It was shown that TORC1 can downregulate Sit4p activity through the phosphorylation of Tap42p, which then inhibits Sit4p [23], a mechanism necessary to activate complex sphingolipid biosynthesis [24, 25].

In this study, we investigated the interplay between these signaling networks in the regulation of acetic acid-induced RCD. We show that Pkh1p-Ypk1p and Pkh1p-Sch9p pathways are activated during acetic acid exposure to induce cell death. In addition, we explored how these signaling pathways affect the resistance phenotype of the *isc1Δ* mutant to acetic acid.

## 2. Material and Methods

### 2.1. Yeast Strains, Plasmids, and Growth Conditions

All *Saccharomyces cerevisiae* strains used in this study are listed in Table 1. The yeast *S*. *cerevisiae* strain BY4741 was used throughout this study as the wild-type strain. The *isc1Δ ypk1Δ* mutant was constructed by homologous recombination of a disruption cassette containing the flanking regions of *YPK1* (*YPK1*::KanMX4), which was amplified by Polymerase Chain Reaction (PCR) using genomic DNA isolated from the *ypk1Δ* mutant, into the BY4741 *isc1Δ*::*LEU2* strain. After transformation using the LiAc method [29], mutants were selected on rich medium (YPD; 1% (*w*/*v*) yeast extract, 2% (*w*/*v*) bactopeptone, and 2% (*w*/*v*) glucose) containing 200 *μ*g/mL geneticin, and proper integration of the cassette was confirmed by PCR. The *isc1Δ pkh1Δ* double mutant was generated by sporulation of the diploids resultant from mating of *pkh1Δ* (*Mat*a) with *isc1Δ* (*Matα*), followed by tetrad dissection by standard techniques.

Strains were grown in Synthetic Complete Galactose medium (SC Gal; 2% (*w*/*v*) Galactose, 0.67% (*w*/*v*) yeast nitrogen base without amino acids, 0.14% (*w*/*v*) drop-out mixture lacking histidine, leucine, tryptophan, and uracil, 0.008% (*w*/*v*) histidine, 0.04% (*w*/*v*) leucine, 0.008% (*w*/*v*) tryptophan, and 0.008% (*w*/*v*) uracil) at 26°C in an orbital shaker at 200 rpm, with a ratio of flask volume/medium of 5 : 1. Strains transformed with the indicated plasmids were grown in the same medium lacking the appropriate amino acids. Galactose was used as the carbon and energy source as it is less effective in the repression of respiratory metabolism, leading to higher mitochondrial mass and facilitating the assessment of the involvement mitochondria in different cell processes [30]. We routinely confirm the same phenotypes in glucose- and galactose-grown cells and have shown in a previous study that translocation of Isc1p to mitochondria in response to acetic acid occurs similarly in glucose- and galactose-grown cells [28].

### 2.2. Cell Death Assays

For acetic acid treatments, exponential phase cells (OD_600_ = 0.2 − 0.5) grown as described before were harvested and suspended in SC Gal at pH 3.0 (set with HCl) without or with 140 mM acetic acid (Panreac, Spain). Cell viability was measured as a percentage of colony-forming units (c.f.u.) on YPD plates.

### 2.3. Assessment of Plasma Membrane Integrity

Plasma membrane integrity was assessed by flow cytometry using propidium iodide (PI, Sigma-Aldrich) staining. PI was added to yeast cell suspensions to a final concentration of 5 *μ*g/mL and incubated for 10 min at room temperature. Cells with red fluorescence (FL-3 channel (488/620 nm)) were considered to have lost their plasma membrane integrity.

### 2.4. Assessment of ROS Accumulation

Intracellular superoxide anion was detected by flow cytometry using dihydroethidium (DHE, Molecular Probes, Eugene, USA). Untreated or acetic acid-treated cells were harvested by centrifugation, suspended in PBS (80 mM Na_2_HPO_4_, 20 mM NaH_2_PO_4_, and 100 mM NaCl), and incubated with 5 *μ*g/mL DHE for 30 min in the dark. Cells with red fluorescence (FL-3 channel (488/620 nm)) were considered to accumulate superoxide anion.

### 2.5. Determination of Oxygen Consumption Rates

Yeast strains were cultured under the conditions described above, harvested, and suspended in water (OD_600nm_ = 20). A Clark electrode connected to a Yellow Spring Instruments 5300 biological oxygen monitor and to a Kipp & Zonen flatbed chart recorder was used. The electrode was immersed in a chamber with 4.6 mL of deionized water stirred with a magnetic bar. The Clark electrode was calibrated at time 0 for 100% oxygen saturation. A volume of 0.4 mL of yeast suspension was added to the chamber, and the oxygen consumption was recorded. The oxygen consumption rate values were calculated from the slopes of the initial part of the oxygen consumption trace and normalized to the dry weight of the respective cell culture.

### 2.6. Determination of Protein Phosphorylation Levels

For phosphorylation detection, cells were grown and treated under the conditions described above, harvested at the indicated times, and processed. Briefly, cells were immediately centrifuged and incubated in 0.25 M NaOH for 5 min at room temperature. After centrifugation, the pellet was suspended in SDS sample buffer (3% (*w*/*v*) DTT, 125 mM Tris, 20% (*v*/*v*) Glycerol, 0.001% (*w*/v) Bromophenol Blue, and 4% (*w*/*v*) SDS) and boiled at 95°C for 5 min. Samples were then separated electrophoretically on a 10% SDS-polyacrylamide gel and transferred to a nitrocellulose membrane (GE Healthcare) at 0.8 mA/cm^2^ for 1 h and 30 min. The membrane was blocked in TBS-T (20 mM Tris, 140 mM NaCl, and 0.05% (*v*/*v*) Tween-20 pH 7.6) containing 5% (*w*/*v*) BSA. Membranes were cut into strips and incubated with the primary antibodies mouse monoclonal anti-yeast phosphoglycerate kinase (PGK1) antibody (1 : 5000, Molecular Probes), rabbit anti-Sch9p antibody (1 : 1000), goat anti-Ypk1p antibody (1 : 100, Santa Cruz Biotechnology), rabbit anti-Phospho-T570-Sch9p antibody (1 : 10000), rabbit anti-Phospho-T504 Ypk1p (1 : 1000, Cell Signaling Technology, Phospho-PKC (pan) (zeta Thr410) (190D10)), and rabbit anti-Phospho-Slt2p (1 : 1000, Cell Signaling, anti-Phospho-p44/42 MAPK (Erk1/2) (Thr202/Tyr204)), followed by incubation with secondary antibodies against mouse, rabbit, or goat IgG peroxidase (1 : 5000; Sigma-Aldrich). Immunodetection of bands was revealed by chemiluminescence (ECL, GE Healthcare).

### 2.7. Immunoprecipitation Analysis

BY4741 cells expressing Isc1p-FLAG or vector control were grown in SC Gal medium without uracil, harvested at early exponential phase, and washed and suspended in IP buffer (0.5% (*v*/*v*) Triton X-100, 150 mM NaCl, 1 mM EDTA, 50 mM Tris pH 7.4, 1x Complete Mini Protease Inhibitor Cocktail (Roche)). Cells were lysed for 10 min (short pulses of 1 min with 1 min intervals on ice) by vigorous shaking of the cell suspension in the presence of glass beads, and total protein concentration was assessed by the Bradford method using bovine serum albumin as a standard. 1000 *μ*g of soluble protein were immunoprecipitated overnight at 4°C with 1 *μ*L of mouse *α*-FLAG M2 antibody (Sigma-Aldrich). Then, 20 *μ*L of Protein G Sepharose (Sigma-Aldrich) were added and the suspension incubated for 2 hours at 4°C, with agitation. The beads were sedimented, and the unbound proteins were removed by three washing steps with IP buffer and collected by centrifugation. Bead-bound immune complexes were solubilized with SDS sample buffer and boiled for 5 min, and the supernatant was analyzed by Western blot as previously described using the primary antibodies goat anti-Ypk1p antibody (1 : 100, Santa Cruz Biotechnology) and mouse anti-FLAG M2 (1 : 5000, Sigma-Aldrich), followed by incubation with secondary antibodies anti-goat IgG-peroxidase (1 : 5000; Sigma-Aldrich) and anti-mouse IgG-peroxidase TrueBlot® ULTRA (1 : 5000; Rockland), respectively.

### 2.8. Cytochrome *c* Release

For cytochrome *c* detection, cells were grown and treated under the conditions described above, harvested at early exponential phase, and suspended in suspension buffer (1.2 M sorbitol, 0.06 M Sodium phosphate (pH 7.5), and 0.01 M EDTA). Cells were then digested with Zymolyase 20 T (ImmunO, MP Biomedicals) to obtain spheroplasts, washed twice with 1.2 M sorbitol, and suspended in lysis buffer (0.5 M Sorbitol, 20 mM Tris/HCl (pH 7.5), and 1 mM EDTA). Spheroplasts were lysed with a few strokes in a glass Dounce homogenizer (tight fitting piston). Homogenates were then centrifuged at 2500 rpm for 10 min, and the supernatant was centrifuged at 15000 rpm for 15 min. The supernatant constitutes the cytosolic fraction. The pellet, containing the mitochondrial fraction, was suspended in lysis buffer and both fractions frozen in liquid nitrogen and stored at -80°C.

Mitochondrial and cytosolic fractions were separated electrophoretically on a 12.5% SDS-polyacrylamide gel and transferred to a Hybond-P polyvinylidene difluoride membrane (PVDF; GE Healthcare) at 0.8 mA/cm^2^ for 1 h. Membranes were blocked for 1 h in PBS-T (PBS with 0.05% (*v*/*v*) Tween-20) containing 5% (*w*/*v*) nonfat dry milk, cut into strips, and incubated with the primary antibodies mouse monoclonal anti-yeast phosphoglycerate kinase (PGK1) antibody (1 : 5000, Molecular Probes), mouse monoclonal anti-yeast porin (POR1) antibody (1 : 10000, Molecular Probes), or rabbit polyclonal anti-yeast cytochrome *c* (CYC1) antibody (1 : 2000, custom-made by MilleGen), followed by incubation with secondary antibodies against mouse or rabbit IgG-peroxidase (1 : 5000; Sigma-Aldrich). Immunodetection of bands was revealed by chemiluminescence (ECL, GE Healthcare).

### 2.9. Calcofluor White Staining

Yeast cells were grown under the conditions described above, harvested at early exponential phase, suspended in PBS, incubated with 0.02% (*w*/*v*) Calcofluor White (CFW, Fluka, St. Louis, MO) for 30 min at room temperature, and washed 3 times with PBS. Cells were observed using a Leica Microsystems DM-5000B epifluorescence microscope with appropriate filter settings using a 100x oil-immersion objective. Images were acquired with a Leica DCF350FX digital camera and processed with LAS AF Leica Microsystems software.

## 3. Results and Discussion

### 3.1. Pkh1p, Ypk1p, and Sit4p Regulate Acetic Acid-Induced Cell Death

**Figure 2:**
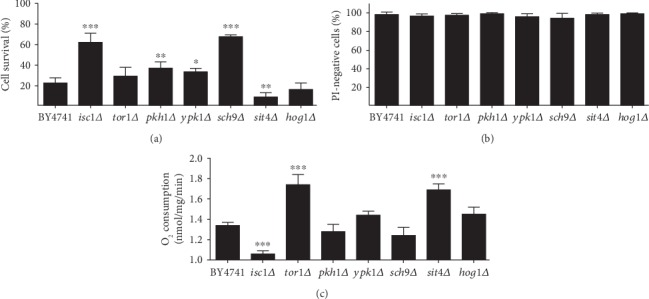
Pkh1p, Ypk1p, Sch9p, and Sit4p regulate acetic acid-induced cell death. (a) Cell survival of the indicated *S. cerevisiae* strains to 140 mM of acetic acid, pH 3.0, for 180 min. Cell viability was determined by standard dilution plate counts and expressed as a percentage of c.f.u. on YPD plates in relation to time 0. (b) Percentage of PI-negative cells of the indicated *S*. *cerevisiae* strains after acetic acid exposure assessed by flow cytometry. (c) Basal O_2_ consumption rates of the indicated *S*. *cerevisiae* strains determined using a Clark electrode. Values represent mean ± SD of at least three independent experiments. Values significantly different from the BY4741 strain: ^∗^*P* < 0.05, ^∗∗^*P* < 0.01, ^∗∗∗^*P* < 0.001, one-way ANOVA, and Tukey's Test.

The involvement of sphingolipid signaling in the regulation of biological processes, such as cell death, has been addressed in both mammals and yeast [4]. In yeast, several signaling pathways have been connected with Isc1p function in the response to several stimuli, like hydrogen peroxide and ageing [31–35]. We therefore aimed to determine the involvement of those signaling pathways in acetic acid-induced RCD. We first assessed the relative contribution of Tor1p, Pkh1p, Ypk1p, Hog1p, and Sit4p to acetic acid-induced RCD under our experimental conditions, and compared with that of Sch9p and Isc1p [28]. For this purpose, *S*. *cerevisiae* BY4741 and *isc1Δ*, *tor1Δ*, *pkh1Δ*, *ypk1Δ*, *sch9Δ*, *sit4Δ*, and *hog1Δ* mutants were grown to exponential phase in SC Gal medium and exposed to 140 mM acetic acid, pH 3.0, for 180 min (Figure 2).

As shown in Figure 2(a), deletion of *PKH1* or *YPK1* increased the resistance of yeast cells to acetic acid, whereas deletion of *SIT4* decreased the resistance and *TOR1* or *HOG1* deletion had no significant effect. Also, in accordance with our previous results, *ISC1* and *SCH9* deletion increased cell survival to a similar extent [28]. Acetic acid-induced cell death in all strains was not associated with significant loss of plasma membrane integrity as measured by propidium iodide (PI) staining, indicating this is a non-necrotic regulated event (Figure 2(b)). Since plasma membrane integrity was assessed after exposing cells to acetic acid for 180 min, but viability assessed only 48 h after plating those cells on rich media, we sought to determine at what point during this period cells died, which ultimately results in plasma membrane disruption. For this purpose, after acetic acid treatment, wild-type cells were washed, suspended in fresh medium, and grown at 26°C. Plasma membrane integrity was again measured by PI staining, and we found that about 90% of wild-type cells were PI positive after 240 min in fresh medium ([Supplementary-material supplementary-material-1]). Our results therefore show that cells that are exposed to acetic acid for 180 min reach the point of no return in the regulated cell death process, still maintaining plasma membrane integrity. Then, even after acetic acid is removed, they continue the cell death process and lose plasma membrane integrity and the ability to form colonies within 240 min.

Cell death induced by acetic acid in *S*. *cerevisiae* is mediated by a mitochondria-dependent pathway and involves ROS accumulation, reduction of oxygen consumption, and translocation of cytochrome *c* from the mitochondria to the cytosol [6]. In addition, respiratory-deficient mutants, namely *ρ*^0^ cells lacking mitochondrial DNA, the null *atp10Δ* mutant (deleted in an assembly factor of mitochondrial ATPase) and the null *cyc3Δ* mutant (deleted in the gene encoding a heme lyase) were described as more resistant to death induced by acetic acid [6]. We therefore sought to determine whether the phenotypes of the mutants described above correlated with changes in mitochondria respiration. For this purpose, cells were grown to exponential phase, and the basal oxygen consumption rate (OCR) was determined using a Clark electrode (Figure 2(c)). The effect of an uncoupler and respiratory chain inhibitors were used as controls to confirm that OCR mirrors mitochondria function ([Supplementary-material supplementary-material-1]). Deletion of *TOR1* or *SIT4* increased the OCR and deletion of *ISC1* decreased OCR, whereas deletion of the other genes had no effect. Indeed, several acetic acid-resistant mutants (*pkh1Δ*, *ypk1Δ*, and *sch9Δ*) exhibited an OCR similar to that of the wild-type. The increase of OCR in *tor1Δ* cells was consistent with previous data showing that disruption of TORC1 signaling, via deletion of *TOR1*, relieves respiration repression by increasing mitochondrial translation, and consequently the number/activity of respiratory complexes [36]. Although Sit4p is negatively regulated by TORC1 signaling [37], previous studies also showed an increase of OCR in *sit4Δ* mutants [31]. Overall, these results suggest that acetic acid resistance of the tested mutants is not associated with basal OCR levels.

In conclusion, besides Sch9p, we identified three new important players of several signaling pathways with a role in acetic acid-induced cell death, namely, Pkh1p and Ypk1p with a pro-death and Sit4p with a pro-survival role.

### 3.2. Acetic Acid Induces Pkh1p-Dependent Phosphorylation of Sch9p and Ypk1p

**Figure 3:**
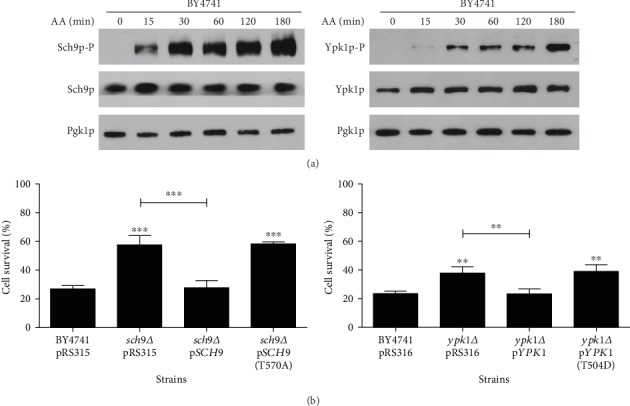
Acetic acid induces PDK1-dependent phosphorylation of Ypk1p and Sch9p. (a) Western blot analysis of the levels of total and phosphorylated Sch9p and Ypk1p in wild-type cells before and after exposure to acetic acid for up to 180 min. Cytosolic phosphoglycerate kinase (Pgk1p) levels were used as the loading control. Only a representative image of Pgk1p levels was represented in the figure, although the levels of Pgk1p were evaluated in both blots of the total and phosphorylated proteins. (b) Cell survival of the indicated strains to 140 mM of acetic acid, pH 3.0, for 180 min. Cell viability was determined by standard dilution plate counts and expressed as a percentage of c.f.u. on YPD plates in relation to time 0. Values represent mean ± SD of at least three independent experiments. Values significantly different from BY4741 or between the indicated strains: ^∗∗^*P* < 0.01, ^∗∗∗^*P* < 0.001, one-way ANOVA, and Tukey's Test.

Given the phenotypic evidence that Pkh1p, Ypk1p, and Sch9p have a pro-death role in acetic acid-induced apoptosis, we next analyzed whether the corresponding signaling pathways were activated. It has been demonstrated that the Pkh1p kinase can differentially activate the Sch9p kinase by phosphorylating its T570 residue [18, 19] or the Ypk1p kinase by phosphorylating its T504 residue [12, 14]. To determine whether Pkh1p activates Ypk1p and/or Sch9p in response to acetic acid, phosphorylated and total protein levels of Sch9p and Ypk1p were analyzed (Figure 3(a)). For this purpose, we used phospho-site-directed antibodies Phospho-PKC (pan) (zeta Thr410) (190D10) directed against the highly homologous PDK1 site in human SGK1, which specifically detects P-Thr504 in Ypk1p [12, 38] and anti-Phospho-T570-Sch9p, which specifically recognizes this PDK1 site in Sch9p [20].

Our data show that acetic acid exposure leads to phosphorylation of both Sch9p and Ypk1p at the PDK1 sites, without affecting the total levels of the corresponding proteins. An increase of phosphorylated Sch9p and Ypk1p was already observed after 15 min, but it further increased with the time of exposure to acetic acid. Phosphorylation of the single PDK1 site in Ypk1p or Sch9p is required for their function *in vivo* [18]. We further confirmed that expression of Sch9p (T570A) and Ypk1p (T504D) does not revert the resistant phenotype of the *sch9Δ* and *ypk1Δ* mutants, respectively, while expression of wild-type Sch9p and Ypk1p renders cells more sensitive to acetic acid, as expected (Figure 3(b)). Though Sch9p can be phosphorylated at T570 by both Pkh1p and Pkh2p, Pkh2p has a minor contribution [20]. It has also been shown that Pkh1p preferentially activates Ypk1p, both biochemically and genetically [16]. Therefore, while we cannot fully exclude a contribution of Pkh2p, our results indicate that Pkh1p phosphorylates Ypk1p and Sch9p to mediate acetic acid-induced RCD.

### 3.3. Pkh1p And Ypk1p Are Necessary for *isc1Δ* Resistance to Acetic Acid-Induced RCD

Our data implicate the Pkh1p-Sch9p and Pkh1p-Ypk1p cascades in acetic acid-induced RCD in the wild-type strain. However, the results suggest that Pkh1p, Ypk1p, and Sch9p may modulate this process in a different manner, as acetic acid resistance of the correspondent mutants was for the most part very different (Figure 2(a)). This could be due to differential functional interplay between the different components of sphingolipid metabolism pathways, and in particular with Isc1p. In line with this, our previous results demonstrated that acetic acid treatment induces Sch9p-dependent translocation of Isc1p from endoplasmic reticulum to mitochondria [28]. However, the higher survival of the *sch9Δ* mutant compared to *pkh1Δ* suggests that Sch9p might also be activated by other kinases different from Pkh1p (possibly Pkh2p and others), and the phosphorylation of Ypk1p by Pkh1/2p suggests that these kinases have other functions independent of Sch9p in acetic acid-induced cell death.

**Figure 4:**
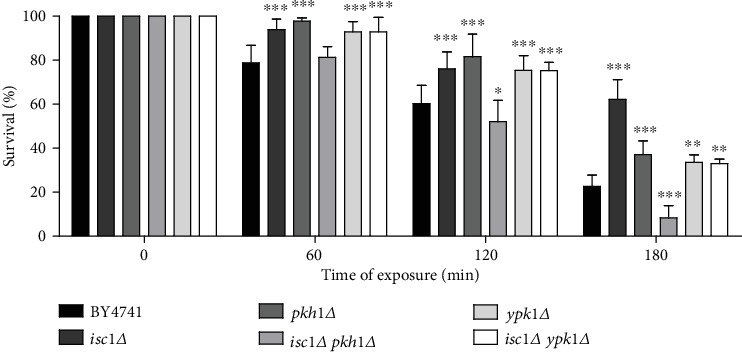
The Pkh1p-Ypk1p pathway is necessary for resistance of the *isc1Δ* mutant to acetic acid-induced RCD. Cell survival of the indicated strains to 140 mM of acetic acid, pH 3.0, for 180 min. Values represent mean ± SD of at least three independent experiments. Values significantly different from BY4741: ^∗^*P* < 0.05, ^∗∗^*P* < 0.01, ^∗∗∗^*P* < 0.001, One-way ANOVA, and Tukey Test.

Isc1p function has been connected with several other signaling pathways involved in ceramide signaling. Indeed, it was demonstrated that deletion of *SIT4* [31], *HOG1* [33], or *TOR1* [35] suppresses the phenotypes displayed by *isc1Δ* cells. Since we found that the Pkh1p-Ypk1p pathway is activated during acetic acid treatment and the survival phenotypes of the mutants were relatively different from *isc1Δ*, we assessed the effect of deleting *PKH1* or *YPK1* in the survival of the *isc1Δ* mutant to acetic acid (Figure 4).

**Figure 5:**
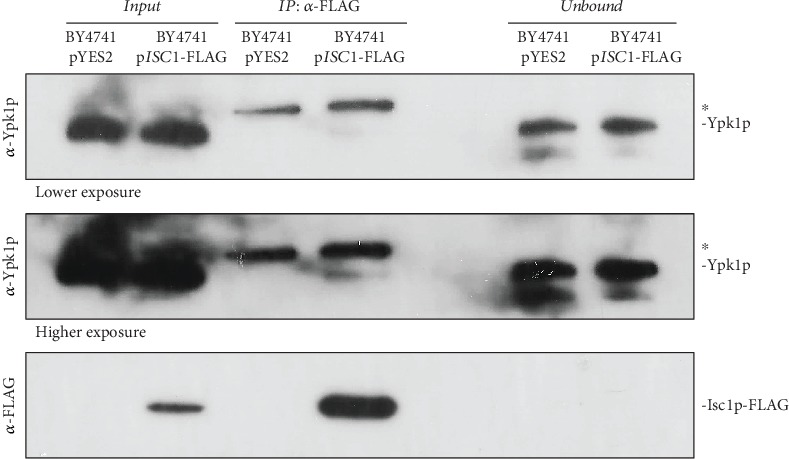
Ypk1p interacts with Isc1p. Isc1p-FLAG was immunoprecipitated from protein extracts of BY4741 cells expressing Isc1p-FLAG or vector control grown in SC Gal medium without uracil to exponential phase, and analyzed by Western blot using anti-Ypk1p and anti-FLAG antibodies. The band marked with an asterisk (^∗^) represents a non-Ypk1p protein that cross-reacts with the anti-Ypk1p antibody. A lower and a higher exposure of the membranes incubated with anti-Ypk1p antibody were displayed to help the visualization of the weak Ypk1p band.

Deletion of either *PKH1* or *YPK1* suppressed the resistant phenotype exhibited by the *isc1Δ* mutant, though to a different extent. Deletion of *YPK1* in the *isc1Δ* mutant decreased cell survival, but the double mutant strain was still more resistant than wild-type cells. This suggests that the resistance of *isc1Δ* cells to acetic acid partially depends on Ypk1p. However, as far as we know, no association had been described between Ypk1p and the yeast neutral sphingomyelinase Isc1p. We therefore sought to determine whether the two proteins physically interact. For this purpose, extracts of BY4741 cells expressing Isc1p-FLAG or vector control were immunoprecipitated with an anti-FLAG antibody, and the presence of Ypk1p in extracts and immunoprecipitated fractions was assessed by Western blot (Figure 5).

We found that Ypk1p can interact with Isc1p, though only a minor amount of total Ypk1p was immunoprecipitated by Isc1p-FLAG, suggesting a weak or transient interaction between the two proteins. This indicates that Ypk1p and Isc1p may function on the same pathway mediating acetic acid-induced cell death.

**Figure 6:**
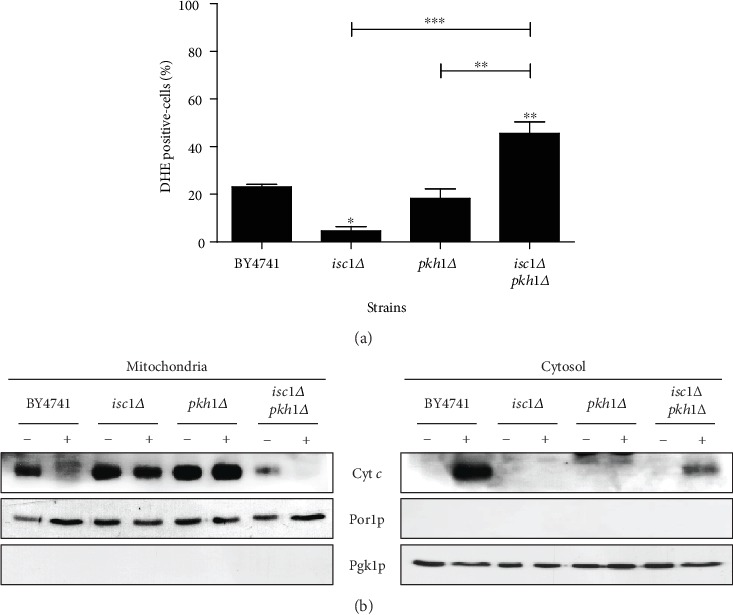
Sensitivity phenotype of the *isc1Δ pkh1Δ* mutant is associated with increased superoxide anion accumulation and release of cytochrome *c*. (a) Levels of superoxide anion in the indicated strains exposed to acetic acid using DHE. (b) Western blot analysis of cytochrome *c* before (-) and after (+) exposure to 140 mM acetic acid, pH 3.0, for 180 min, in both mitochondrial and cytosolic fractions. Cytosolic phosphoglycerate kinase (Pgk1p) and mitochondrial porin (Por1p) levels were used as loading control of cytosolic and mitochondrial fractions, respectively. In (a), values represent mean ± SD of at least three independent experiments. Values significantly different from BY4741 or between the indicated strains: ^∗^*P* < 0.05, ^∗∗^*P* < 0.01, ^∗∗∗^*P* < 0.001, one-way ANOVA, and Tukey's Test.

On the other hand, double deletion of *ISC1* and *PKH1* had a surprising drastic effect on cell survival, i.e., the *isc1Δ pkh1Δ* double mutant was much more sensitive to acetic acid than the wild-type strain, even though the single mutants were resistant. We therefore characterized the apoptotic phenotype of *isc1Δ pkh1Δ* cells (Figures 6(a) and 6(b)).

We observed that the high sensitivity phenotype of the double mutant was associated with increased superoxide anion accumulation and release of cytochrome *c* that was not observed in the single-mutant strains. Therefore, simultaneous absence of *PKH1* and *ISC1* seemingly overrides the protective effect as a result of individual deletion of *PKH1* or *ISC1.* As we previously showed [11], we observed that the absence of Isc1p led to impaired cytochrome *c* release, and we further show here that the absence of Pkh1p results in the same phenotype. This suggests that Isc1p and Pkh1p are required for cytochrome *c* release. However, since *pkh1Δ isc1Δ* cells can release cytochrome *c*, it is apparent that there is not an absolute requirement of Isc1p or Pkh1p for cytochrome *c* release, and therefore, the lack of cytochrome *c* release in individual mutants may be a consequence and not a cause of the increased resistance of those cells. The increased sensitivity of *isc1Δ pkh1Δ* cells was nonetheless unexpected, and we therefore investigated the upstream events leading to this phenotype.

### 3.4. CWI Signaling Pathway Activation Is Not Involved in the Sensitivity of *isc1Δ pkh1Δ* Cells to Acetic Acid-Induced RCD

During growth and in response to environmental stresses, the cell wall is remodeled in a highly regulated process under the control of the CWI signaling pathway, which facilitates the maintenance of the cell wall by mediating cell wall biosynthesis, actin organization, and other cellular processes [39]. Both the Pkh1p-Ypk1p pathway and Isc1p were previously implicated in CWI signaling. It was shown that Pkh1p phosphorylates Pkc1p, a protein kinase C-like enzyme member of CWI pathway [14], and Ypk1p may contribute to Slt2p activation, leading to activation of the pathway [40]. Additionally, it was found that Isc1p-deficient cells displayed Slt2p hyperphosphorylation and that Slt2p is essential for viability of *isc1Δ* cells, since deletion of *SLT2* in this mutant results in synthetic lethality [33]. The relation of both Pkh1p and Isc1p with the CWI pathway led us to investigate whether the sensitivity phenotype of the *isc1Δ pkh1Δ* double mutant could be due to deregulation in the CWI pathway leading to cell wall defects and consequently cell death.

**Figure 7:**
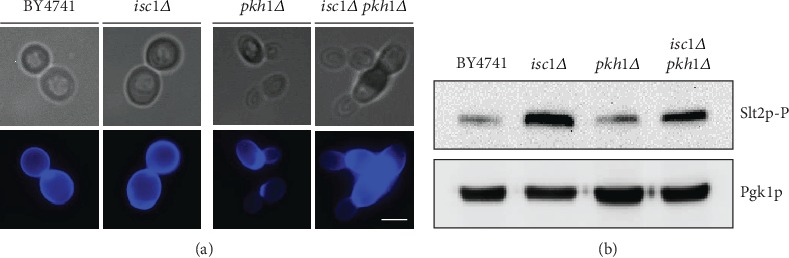
*PKH1* deletion increases cell wall defects in the *isc1Δ* mutant strain but does not increase its levels of Slt2p phosphorylation. (a) Cell wall morphology of the indicated strains stained with CFW and observed by fluorescence microscopy. Representative images of two independent experiments are shown. Bar, 5 *μ*m. (b) Western blot analysis of the basal levels of phosphorylated Slt2p in the indicated strains.

To test this hypothesis, the mutant strains were grown to exponential phase in SC Gal medium and stained with Calcofluor White (CFW) (Figure 7(a)). CFW is a fluorescent stain that binds to structures containing chitin like the yeast cell wall and has been widely used to identify strains with cell wall defects [41]. The CFW stain revealed the presence of chitin in cell walls and its normal accumulation at the bud sites. The morphology of the *isc1Δ* and *pkh1Δ* strain was quite similar to that of the wild-type strain; however, deletion of both *ISC1* and *PKH1* resulted in the formation of aberrant cells with elongated buds, wide necks, and large accumulation of chitin in the cells.

The Slt2p MAP kinase is activated by phosphorylation in response to cell wall perturbations. Consistently, our previous results indicated that the CWI pathway is involved in signaling acetic acid-induced cell death, as blocking signal transduction through this pathway renders cells more resistant to acetic acid-induced cell death, and overactivation of the CWI renders cells more sensitive to acetic acid [42]. We therefore investigated whether the high sensitivity of the *isc1Δ pkh1Δ* double mutant to acetic acid resulted from overactivation of the CWI pathway. As seen in Figure 7(b), Isc1p-deficient cells displayed Slt2p hyperphosphorylation as described before [33]. However, despite the basal cell wall defects observed in the *isc1Δ pkh1Δ* double mutant, we did not observe an increased level of Slt2p phosphorylation in comparison with the *isc1Δ* mutant. Thus, both the high resistance and high sensitivity to acetic acid of the *isc1Δ* and *isc1Δ pkh1Δ* double mutants, respectively, are not associated with CWI signaling.

### 3.5. The Sensitivity of *isc1Δ pkh1Δ* to Acetic Acid-Induced RCD Depends on cAMP Signaling

**Figure 8:**
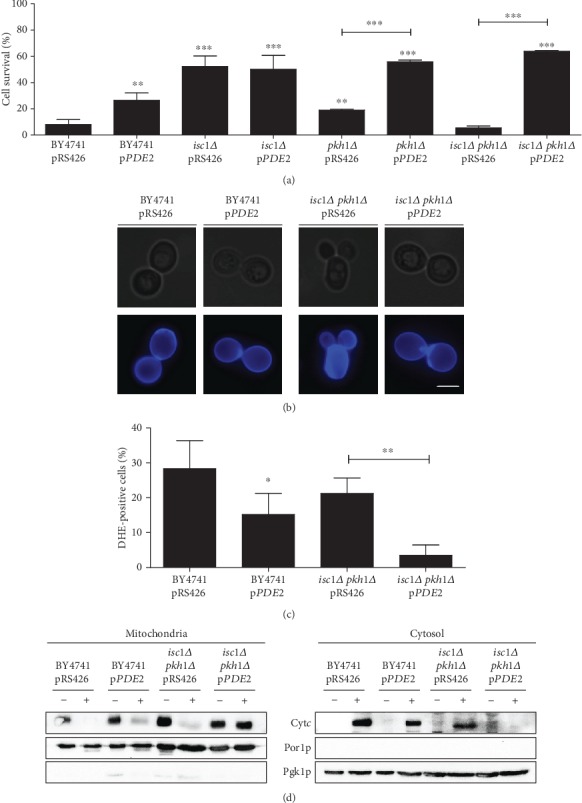
cAMP/PKA pathway is involved in the sensitivity of the *isc1Δ pkh1Δ* mutant to acetic acid-induced apoptosis. (a) Cell survival of the indicated strains to 140 mM of acetic acid, pH 3.0, for 180 min. (b) Cell wall morphology of the indicated strains stained with CFW and observed by fluorescence microscopy. Representative images of two independent experiments are shown. Bar, 5 *μ*m. (c) Levels of superoxide anion in the indicated strains exposed to acetic acid using DHE. (c) Western blot analysis of cytochrome *c* before (-) and after (+) exposure to 140 mM acetic acid, pH 3.0, for 180 min, in both mitochondrial and cytosolic fractions. Cytosolic phosphoglycerate kinase (Pgk1p) and mitochondrial porin (Por1p) levels were used as loading control of cytosolic and mitochondrial fractions, respectively. In (a) and (c), values represent mean ± SD of at least three independent experiments. Values significantly different from BY4741 pRS426 or between the indicated strains: ^∗^*P* < 0.05, ^∗∗^*P* < 0.01, ^∗∗∗^*P* < 0.001, one-way ANOVA, and Tukey's Test.

The maintenance of the intracellular pH and the development of a stress response are crucial for yeast cells to survive in an acidic environment. At low pH, acetic acid (undissociated form) enters cells by simple diffusion when grown under glucose-repressed or non-acetic acid permease induction conditions [43]. Another study proposes that the aquaglyceroporin channel Fps1p mediates the transport through a facilitated diffusion process [44]. In any case, once in the cytoplasm and due to a more neutral pH, acetic acid dissociates into acetate and protons, leading to cytoplasmic acidification and inhibition of important metabolic processes which include redox homeostasis, enzymatic activities, or nutrient transport [45–48]. Intracellular acidification has also been described to stimulate the Ras/cAMP/PKA pathway [49, 50]. The levels of cAMP depend on both its synthesis (Ras/adenylate cyclase module) and degradation (cAMP phosphodiesterases) [51]. The yeast cAMP phosphodiesterases Pde1p and Pde2p have low and a high affinity for cAMP, respectively, inhibit PKA by hydrolyzing cAMP and have been shown to be involved in the ability of yeast cells to survive stress conditions [52–54]. Consistently, both cytoplasmic acidification and Ras/cAMP/PKA pathway activation have been described to occur during acetic acid-induced cell death in yeast [7, 55]. For this reason, we questioned whether the sensitivity phenotype of the *isc1Δ pkh1Δ* double mutant could be due to a hyperactivation of the cAMP/PKA pathway in this strain. To test this hypothesis, the wild-type and mutant strains were transformed with a plasmid driving overexpression of *PDE2* (pRS426-*PDE2*) or the empty plasmid (pRS426), grown to early exponential phase in SC Gal medium without uracil, exposed to 140 mM acetic acid, pH 3.0, for 180 min, and characterized regarding acetic acid-induced cell death (Figure 8).

As seen in Figure 8(a), wild-type cells overexpressing *PDE2* showed enhanced resistance to acetic acid in comparison with the control strain. In contrast, *PDE2* overexpression had no effect on the survival of the *isc1Δ* mutant. As observed for the wild-type strain, overexpression of *PDE2* increased the survival of *pkh1Δ* and *isc1Δ pkh1Δ* mutant cells to acetic acid to levels observed in *isc1Δ* mutants. The observation that Pde2p overexpression completely suppresses the sensitivity phenotype of the *isc1Δ pkh1Δ* double mutant suggests that a hyperactivation of the cAMP/PKA pathway underlies the sensitivity of this strain to acetic acid. Since inappropriate hyperactivation of the cAMP/PKA pathway was also shown to lead to mitochondrial dysfunction, superoxide anion accumulation, and cell death [56], we characterized the phenotype of these cells in more detail (Figures 8(b)–8(d)). We found that expression of *PDE2* in the *isc1Δ pkh1Δ* double mutant abolished the formation of aberrant cells and large accumulation of chitin observed in *isc1Δ pkh1Δ* cells, and decreased the superoxide anion accumulation and cytochrome *c* release in response to acetic acid. These results indicate that Pkhp1 and Isc1p regulate cAMP levels through a yet uncharacterized mechanism and that the increased sensitivity of *isc1Δ pkh1Δ* cells to acetic acid is due to abnormally increased cAMP levels.

## 4. Conclusions

In this study, the relative contribution of several signaling pathways and how sphingolipid signaling regulates acetic acid-induced cell death, possibly through Isc1p as a downstream target, was determined. We first showed that Pkh1p and Ypk1p have a pro-death role, similarly to Isc1p and Sch9p [28]. We also found that acetic acid exposure leads to Pkh1/2p-dependent phosphorylation of both Sch9p and Ypk1p, indicating that Pkh1p-Ypk1p and Pkh1p-Sch9p pathways are activated during acetic acid-induced cell death. Since we had previously described that Sch9p is involved in Isc1p translocation to mitochondria, the role of the Pkh1p-Ypk1p pathway in the resistance of *isc1Δ* was addressed in more detail. We show that deletion of either *YPK1* or *PKH1* leads to a decrease in the survival of *isc1Δ* mutants exposed to acetic acid. However, while *isc1Δ ypk1Δ* cells were still more resistant than wild-type, double deletion of *PKH1* and *ISC1* resulted in hypersensitivity, leading to the emergence of apoptotic markers such as superoxide anion accumulation and cytochrome *c* release. This was correlated with presumed increased cAMP levels, as it was reverted by overexpression of Pde2p. Indeed, the resistant phenotypes of single (*isc1Δ* or *pkh1Δ*) and the double (*isc1Δ pkh1Δ*) mutants overexpressing Pde2p were very similar, which indicates that Pkh1p and Isc1p play a role in the mediation of acetic acid-induced cell death under those conditions. Therefore, the sensitive phenotype of *isc1Δ pkh1Δ* cells is likely an indirect consequence of increased cAMP levels, which leads to acetic acid sensitivity, and not a result of a direct protective role of either Pkh1p or Isc1p in acetic acid-induced cell death.

Taken together, our results suggest that both Pkh1p-Ypk1p and Pkh1p-Sch9p pathways play a role in Isc1p-mediated acetic acid-induced cell death. Sch9p seems to be important for Isc1p activation and translocation into mitochondria [28]. As we observed that Ypk1p may interact with Isc1p, it will be interesting in the future to determine whether Ypk1p and/or Pkh1p are involved in translocation of Isc1p, as observed for Sch9p [28]. On the other hand, it has been reported that Ypk1p is responsible for the phosphorylation of Lac1p and Lag1p ceramide synthases [26]. Therefore, the Pkh1p-Ypk1p pathway might also be involved in the activation of Lag1p in acetic acid-induced cell death in wild-type cells. In accordance with the hypothesis that Ypk1p activates Lag1p, we have previously shown that the deletion of *LAG1* increases the cellular resistance of yeasts to acetic acid-induced cell death [11]. Another study on acetic acid tolerance already showed that Ypk1p, being activated by Torc2p, phosphorylates Lag1p and Lac1p [57]. Further studies to decipher the link between the Pkh1p-Ypk1p pathway and ceramide *de novo* biosynthesis should be performed.

Overall, our results suggest that Pkh1p-Ypk1p and Pkh1p-Sch9p pathways are activated during acetic acid-induced cell death. Since the importance of ceramide in mammalian cell death is increasingly apparent, our study further supports the use of yeast as a valuable model to investigate the modulation of regulated cell death by ceramide.

## Figures and Tables

**Table 1:** List of *S*. *cerevisiae* strains used in this study.

Strain	Genotype	Source
BY4741	*MAT*a, *his3-1*, *leu2-0*, *met15-0*, *ura3-0*	Euroscarf
BY4741 pYES2	BY4741 harboring pYES2	This study
BY4741 p*ISC1*-FLAG	BY4741 harboring pYES2-*ISC1*-FLAG	This study
BY4741 pRS315	BY4741 harboring pRS315	This study
BY4741 pRS316	BY4741 harboring pRS316	This study
BY4741 pRS426	BY4741 harboring pRS426	This study
BY4741 p*PDE2*	BY4741 harboring pRS426-*PDE2*	This study
*isc1Δ*	BY4741 *isc1Δ*::*LEU2*	[31]
*isc1Δ* pRS426	*isc1Δ* harboring pRS426	This study
*isc1Δ* p*PDE2*	*isc1Δ* harboring pRS426-*PDE2*	This study
*tor1Δ*	BY4741 *tor1Δ*::*KanMX4*	Euroscarf
*pkh1Δ*	BY4741 *pkh1Δ*::*KanMX4*	Euroscarf
*pkh1Δ* pRS426	*pkh1Δ* harboring pRS426	This study
*pkh1Δ* p*PDE2*	*pkh1Δ* harboring pRS426-*PDE2*	This study
*isc1Δ pkh1Δ*	BY4741 *isc1Δ*::*LEU2 pkh1Δ*::*KanMX4*	This study
*isc1Δ pkh1Δ* pRS426	*isc1Δ pkh1Δ* harboring pRS426	This study
*isc1Δ pkh1Δ* p*PDE2*	*isc1Δ pkh1Δ* harboring pRS426-PDE2	This study
*ypk1Δ*	BY4741 *ypk1Δ*::*KanMX4*	Euroscarf
*ypk1Δ* pRS316	*ypk1Δ* harboring pRS316	This study
*ypk1Δ* p*YPK1*	*ypk1Δ* harboring pRS316-*YPK1*	This study
*ypk1Δ* p*YPK1*(T504D)	*ypk1Δ* harboring pRS316-*YPK1*(T504D)	This study
*isc1Δ ypk1Δ*	BY4741 *isc1Δ*::*LEU2 ypk1Δ*::*KanMX4*	This study
*sch9Δ*	BY4741 *sch9Δ*::*KanMX4*	Euroscarf
*sch9Δ* pRS315	*sch9Δ* harboring pRS315	This study
*sch9Δ* p*SCH9*	*sch9Δ* harboring pRS315-*SCH9*	This study
*sch9Δ* p*SCH9*(T570A)	*sch9Δ* harboring pRS315-*SCH9*(T570A)	This study
*sit4Δ*	BY4741 *sit4Δ*::*KanMX4*	Euroscarf
*hog1Δ*	BY4741 *hog1Δ*::*KanMX4*	Euroscarf

## Data Availability

The data used to support the findings of this study are included within the article and the supplementary information file.

## Supplementary Materials

Supplementary Materials Figure S1: percentage of PI-positive wild-type cells after 180 min of acetic acid exposure (treatment) and 120 min and 240 min after transfer to fresh medium. BY4741 cells were grown in Synthetic Complete Galactose (SC Gal) medium to early exponential phase at 26°C and 200 rpm, harvested, and suspended in SC Gal at pH 3.0 without or with 140 mM acetic acid (AA). After 180 min of treatment, plasma membrane integrity was assessed by flow cytometry using propidium iodide (PI) staining. PI was added to yeast cell suspensions to a final concentration of 5 *μ*g/mL and incubated for 10 min at room temperature. Next, the remaining cells were harvested, washed, suspended in fresh SC Gal medium, and incubated at 26°C and 200 rpm. After 120 min and 240 min, plasma membrane integrity was assessed as described before. Values represent mean ± SD of at least three independent experiments. Values significantly different from the BY4741 control strain: ^∗∗∗^*P* < 0.001, One-way ANOVA, and Tukey Test. Figure S2: basal O2 consumption rates of the of wild-type strain in the absence and presence of the uncoupler Carbonyl Cyanide-p-trifluoromethoxyphenylhydrazone (FCCP) and the respiratory chain inhibitors sodium azide and Antimycin A. BY4741 cells were grown in Synthetic Complete Galactose (SC Gal) medium to early exponential phase at 26°C and 200 rpm, then harvested, and suspended in water (OD_600nm_ = 20). A volume of 0.4 mL of yeast suspension was added to the chamber with 4.6 mL of deionized water and stirred with a magnetic bar, and the oxygen consumption was recorded with a Clark electrode. FCCP and sodium azide were added to yeast cell suspensions in the chamber to a final concentration of 1 and 5 *μ*M, and Antimiycin A to 0.5 and 1 nM. The oxygen consumption rate values were calculated from the slopes of the initial part of the oxygen consumption trace and normalized to the dry weight of the cell culture. Values represent mean ± SD of at least three independent experiments. Values significantly different from the BY4741 control strain: ^∗∗∗^*P* < 0.001, one-way ANOVA, and Tukey's Test.
