# Supplementary material for: Pkh1p-Ypk1p and Pkh1p-Sch9p Pathways Are Activated by Acetic Acid to Induce a Mitochondrial-Dependent Regulated Cell Death
Source: Oxid Med Cell Longev. 2020 Apr 2;2020:7095078. doi: 10.1155/2020/7095078 (PMC7154982; doi:10.1155/2020/7095078)
Supplement: Supplementary Materials — Figure S1: percentage of PI-positive wild-type cells after 180 min of acetic acid exposure (treatment) and 120 min and 240 min after transfer to fresh medium. BY4741 cells were grown in Synthetic Complete Galactose (SC Gal) medium to early exponential phase at 26°C and 200 rpm, harvested, and suspended in SC Gal at pH 3.0 without or with 140 mM acetic acid (AA). After 180 min of treatment, plasma membrane integrity was assessed by flow cytometry using propidium iodide (PI) staining. PI was added to yeast cell suspensions to a final concentration of 5 μg/mL and incubated for 10 min at room temperature. Next, the remaining cells were harvested, washed, suspended in fresh SC Gal medium, and incubated at 26°C and 200 rpm. After 120 min and 240 min, plasma membrane integrity was assessed as described before. Values represent mean ± SD of at least three independent experiments. Values significantly different from the BY4741 control strain: ∗∗∗P < 0.001, One-way ANOVA, and Tukey Test. Figure S2: basal O2 consumption rates of the of wild-type strain in the absence and presence of the uncoupler Carbonyl Cyanide-p-trifluoromethoxyphenylhydrazone (FCCP) and the respiratory chain inhibitors sodium azide and Antimycin A. BY4741 cells were grown in Synthetic Complete Galactose (SC Gal) medium to early exponential phase at 26°C and 200 rpm, then harvested, and suspended in water (OD600nm = 20). A volume of 0.4 mL of yeast suspension was added to the chamber with 4.6 mL of deionized water and stirred with a magnetic bar, and the oxygen consumption was recorded with a Clark electrode. FCCP and sodium azide were added to yeast cell suspensions in the chamber to a final concentration of 1 and 5 μM, and Antimiycin A to 0.5 and 1 nM. The oxygen consumption rate values were calculated from the slopes of the initial part of the oxygen consumption trace and normalized to the dry weight of the cell culture. Values represent mean ± SD of at least three independent experiments. Values signifi [file 7095078.f1.pdf]

Supplementary Materials

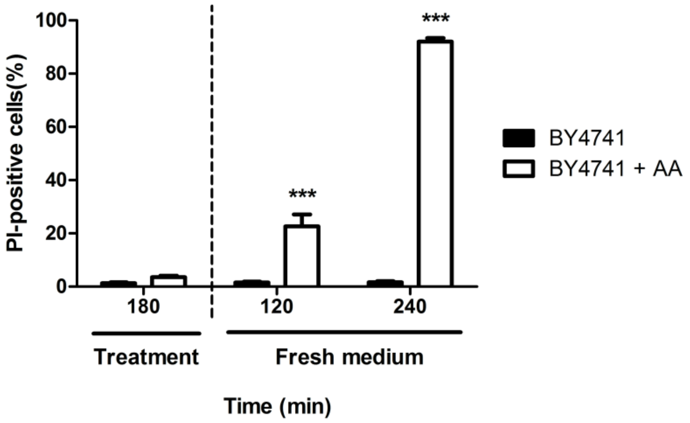

Figure S1 - Percentage of PI-positive wild-type cells after 180 min of acetic acid exposure (treatment), and 120 min and 240 min after transfer to fresh medium. BY4741 cells were grown in Synthetic Complete Galactose (SC Gal) medium to early exponential phase at 26ºC and 200 rpm, harvested and suspended in SC Gal at pH 3.0 without or with 140 mM acetic acid (AA). After 180 min of treatment, plasma membrane integrity was assessed by flow cytometry using propidium iodide (PI) staining. PI was added to yeast cell suspensions to a final concentration of 5 µg/mL and incubated for 10 min at room temperature. Next, the remaining cells, were harvested, washed, suspended in fresh SC Gal medium and incubated at 26ºC and 200 rpm. After 120 min and 240 min, plasma membrane integrity was assessed as described before. Values represent mean ± SD of at least three independent experiments. Values significantly different from the BY4741 control strain: *** $P < 0.001$, One-way ANOVA and Tukey Test.

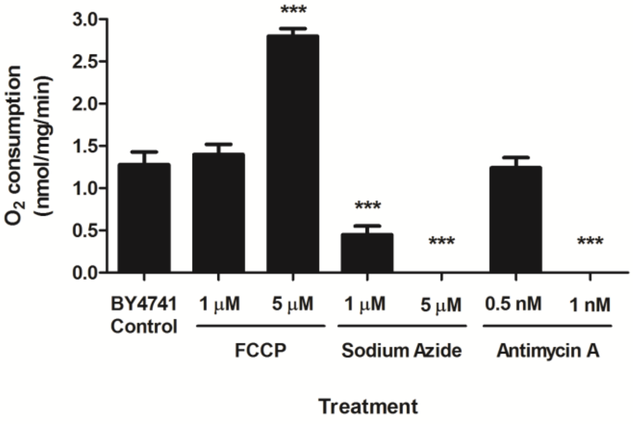

Figure S2 - Basal O_2 consumption rates of the of wild-type strain in the absence and presence of the uncoupler Carbonyl Cyanide-p-trifluoromethoxyphenylhydrazone (FCCP) and the respiratory chain inhibitors sodium azide and Antimycin A. BY4741 cells were grown in Synthetic Complete Galactose (SC Gal) medium to early exponential phase at 26ºC and 200 rpm, then harvested and suspended in water (OD_{600nm} = 20). A volume of 0.4 mL of yeast suspension was added to the chamber with 4.6 mL of deionized water, stirred with a magnetic bar and the oxygen consumption was recorded with a Clark electrode. FCCP and sodium azide were added to yeast cell suspensions in the chamber to a final concentration of 1 and 5 µM, and Antimiycin A to 0.5 and 1 nM. The oxygen consumption rate values were calculated from the slopes of the initial part of the oxygen consumption trace, and normalized to the dry weight of the cell culture. Values represent mean ± SD of at least three independent experiments. Values significantly different from the BY4741 control strain: *** $P < 0.001$, One-way ANOVA and Tukey Test.
